# Recurrent Hematuria Unmasking a Rare Case of Bladder Schistosomiasis in a Child Living in a Non-endemic Country: A Case Report

**DOI:** 10.7759/cureus.100662

**Published:** 2026-01-03

**Authors:** Hany M Elkordy, Mohamed Elkordi, Greta Peciulyte, Saad Mina

**Affiliations:** 1 Urology, Prime Healthcare Group, Dubai, ARE; 2 Urology, CosmesSurge-New Medical Centre (NMC) Healthcare, Abu Dhabi, ARE; 3 Urology, L’Homme Inti Clinic, Premier Medical Services Ltd, London, GBR; 4 Urology, Tanta Model Medical Complex Hospital, Health Insurance Organization, Tanta, EGY

**Keywords:** bladder schistosomiasis, cystoscopy, non-endemic region, pediatric hematuria, praziquantel treatment, schistosoma haematobium, urine cytology

## Abstract

Bladder schistosomiasis is a parasitic infection rarely encountered in non-endemic regions. We report the case of an 11-year-old girl living in the United Arab Emirates (UAE) who presented with recurrent, painless gross hematuria. Urine cytology revealed *Schistosoma haematobium* eggs, and imaging demonstrated bladder wall calcifications. Cystoscopy showed extensive sandy patches, and histopathology confirmed the diagnosis. The patient was treated with praziquantel and achieved complete clinical and radiological resolution. This case highlights the importance of considering schistosomiasis in the differential diagnosis of unexplained hematuria, even in non-endemic regions, particularly in patients with relevant travel history. It underscores the need for heightened awareness of parasitic causes of hematuria in the context of global migration and travel.

## Introduction

Schistosomiasis is a neglected tropical disease caused by parasitic flatworms of the *Schistosoma *species, affecting individuals primarily in endemic regions. It is classified into urogenital and intestinal forms depending on the infecting species. *Schistosoma haematobium* is the predominant cause of urogenital schistosomiasis, affecting the bladder and urinary tract, and may lead to hematuria, chronic inflammation, fibrosis, bladder wall calcification, and even malignancy [1]. According to the World Health Organization, more than 200 million individuals are infected worldwide [2], although urogenital schistosomiasis has become exceedingly rare in the Arabian Peninsula due to improved sanitation and eradication programs. In non-endemic settings such as the United Arab Emirates (UAE), imported cases are typically seen in expatriate workers or travelers returning from endemic regions. Delayed diagnosis can result in unnecessary antibiotic use and progression to irreversible bladder damage. This report describes the case of an 11-year-old girl diagnosed with bladder schistosomiasis in the UAE, emphasizing the importance of travel history and early recognition in the diagnostic process [3]. 

## Case presentation

An 11-year-old female patient from Kenya presented to the urology clinic with a six-month history of recurrent painless gross hematuria without clots, associated with storage symptoms including urinary frequency and occasional urgency. She had no relevant medical or surgical history and was otherwise healthy. Although she was born and residing in the UAE, her family reported occasional short visits to Kenya (approximately once every one to two years for brief stays), indicating limited but notable exposure risk in an endemic environment. 

Physical examination revealed a hemodynamically stable, afebrile child with a soft, non-tender abdomen and no palpable masses. Laboratory investigations, including complete blood count, renal profile, and electrolytes, were within normal limits, with no eosinophilia or anemia. Urinalysis demonstrated numerous red blood cells and elevated white blood cells, while urine culture demonstrated no bacterial growth, indicating sterile pyuria (Table 1).

**Table 1:** Baseline laboratory investigations summarizing the patient’s hematological, biochemical, and urine analysis findings at the time of presentation. The results demonstrate significant microscopic hematuria and pyuria, while renal function and serum electrolytes were within normal limits for age. HPF, high-power field; g/dL, grams per deciliter; mg/dL, milligrams per deciliter; mmol/L, millimoles per liter; ×10⁹/L, ×10 to the power of 9 per liter

Laboratory parameter	Result	Reference range	Unit
Hemoglobin	11.5	10.8–15.6	g/dL
White blood cell (WBC) count	5.77	5.0–13.0	×10⁹/L
Platelet count	279	170–450	×10⁹/L
Eosinophils	4.5	1–8	%
Serum creatinine	0.47	0.4–0.7	mg/dL
Serum urea	20	15–38	mg/dL
Serum sodium	138	136–146	mmol/L
Serum potassium	4.0	3.5–5.1	mmol/L
Urine WBCs	11–15	< 5	cells/HPF
Urine red blood cells (RBCs)	>100	< 3	cells/HPF

Urine cytology was performed early due to persistent painless hematuria despite negative cultures, to evaluate for rare etiologies such as parasitic ova or malignant cells. Cytology revealed *Schistosoma haematobium* eggs in a hemorrhagic and suppurative background with no malignant features. 

**Figure 1:**
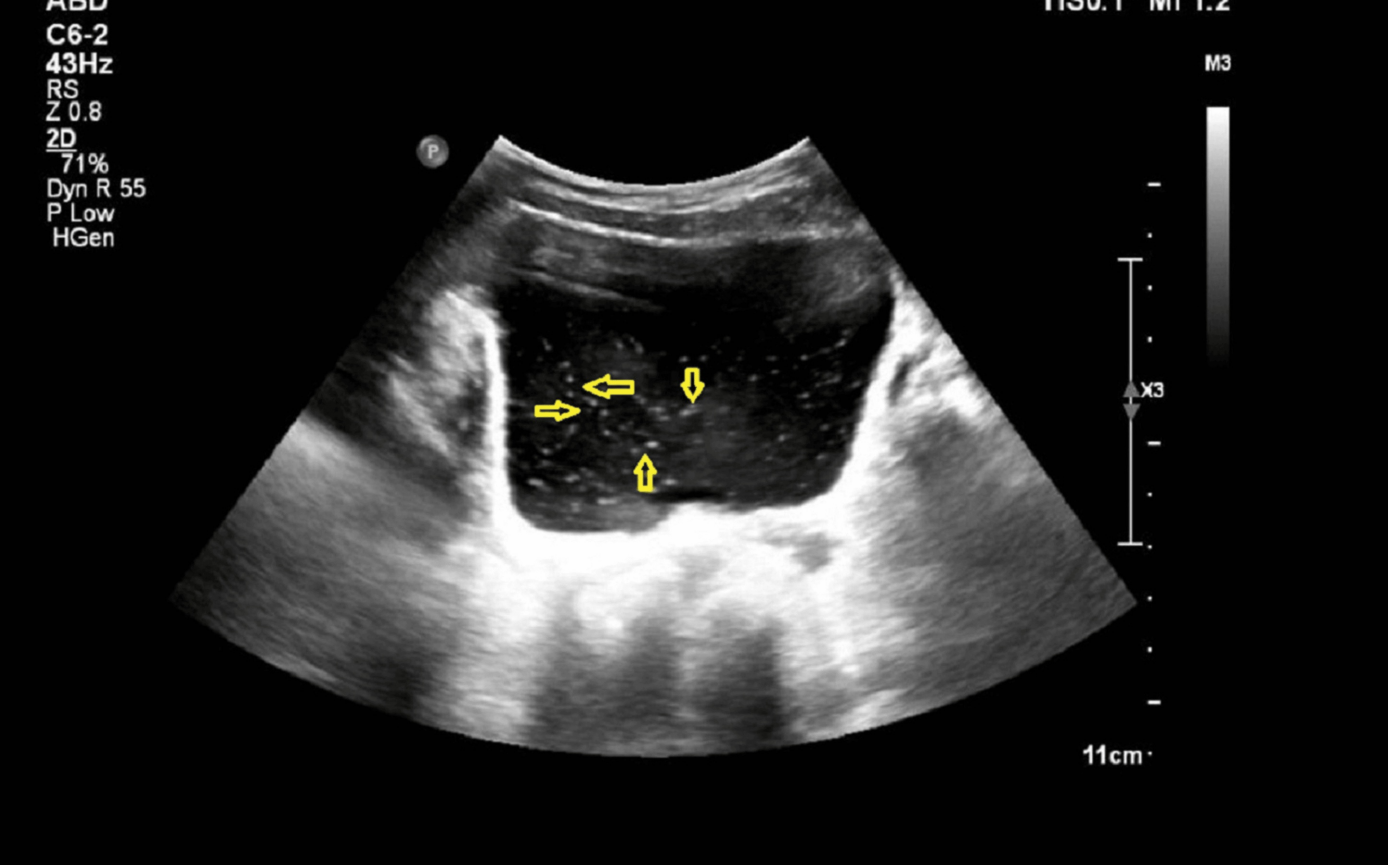
Ultrasound of the kidneys, ureters, and bladder (KUB) revealed floating intraluminal echoes within the urinary bladder (yellow arrows).

Ultrasound of the kidneys, ureters, and bladder (KUB) revealed floating intraluminal echoes within the urinary bladder (Figure 1).

**Figure 2:**
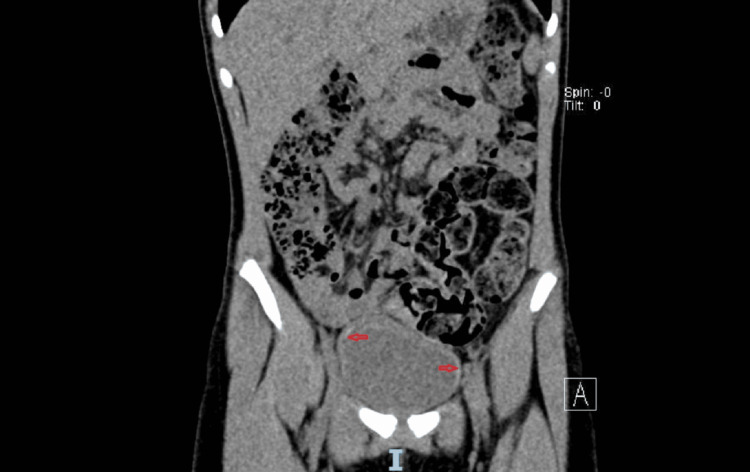
CT urography demonstrates thin, patchy calcifications along the inner bladder wall, consistent with submucosal calcifications (red arrows).

CT urography demonstrated thin, patchy calcifications along the inner bladder wall consistent with submucosal calcifications (Figure 2).

**Figure 3:**
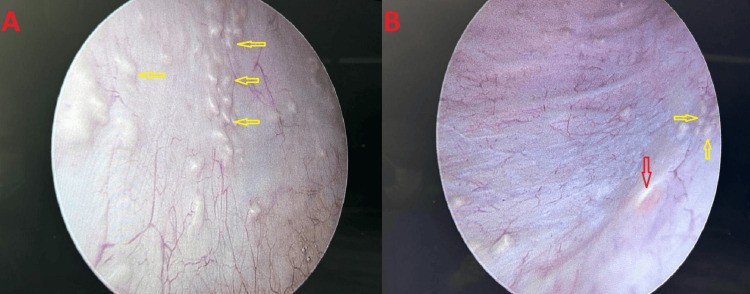
Cystoscopic findings in the present case of bladder schistosomiasis. (A) Cystoscopic view of the posterior bladder wall mucosa demonstrating multiple characteristic sandy patches (yellow arrows) consistent with calcified schistosomal egg deposition. (B) Cystoscopic view of the left lateral bladder wall showing sandy patches (yellow arrows) adjacent to the left ureteric orifice (red arrow), with partial visualization of the trigone.

Cystourethroscopy was performed under general anesthesia and revealed extensive sandy patches covering the bladder mucosa. Cold-cup biopsies were obtained from both lateral bladder walls, the posterior bladder wall, and the trigone (Figure 3).

Histopathology demonstrated preserved urothelial morphology with *Schistosoma haematobium *ova associated with a giant-cell reaction, with no evidence of dysplasia or malignancy. 

The patient was treated with praziquantel at a single oral dose of 40 mg/kg, repeated after four weeks. Treatment was well-tolerated without adverse effects. At six-month follow-up, she was asymptomatic, and repeat urinalysis and urine cytology showed no *Schistosoma haematobium* ova. Ultrasound KUB was normal, and renal function remained stable (Table 2).

**Table 2:** Follow-up laboratory investigations after treatment demonstrating normalization of urine red blood cells and white blood cells, with stable hematological indices and preserved renal function. HPF, high-power field; g/dL, grams per deciliter; mg/dL, milligrams per deciliter; ×10⁹/L, ×10 to the power of 9 per liter.

Laboratory parameter	Result	Reference range	Unit
Hemoglobin	11.2	10.8–15.6	g/dL
White blood cell (WBC) count	8.7	5.0–13.0	×10⁹/L
Platelet count	271	170–450	×10⁹/L
Eosinophils	0.8	1–8	%
Serum creatinine	0.44	0.4–0.7	mg/dL
Serum urea	24	15–38	mg/dL
Urine WBCs	0–2	< 5	cells/HPF
Urine red blood cells (RBCs)	0–2	< 3	cells/HPF

The family received education about schistosomiasis transmission and prevention. She was placed on long-term surveillance with periodic urinalysis and imaging due to the risk of delayed complications in chronic schistosomiasis. 

## Discussion

According to the World Health Organization, bladder schistosomiasis, caused by *Schistosoma haematobium*, remains endemic across several regions of sub-Saharan Africa and the Middle East, including Kenya, Sudan, Tanzania, Uganda, Zimbabwe, Zambia, Togo, Eswatini, Mozambique, and Yemen [4]. Transmission occurs through exposure to freshwater containing infected snails that release cercariae, which penetrate the human skin and then migrate and mature in the circulatory system into adult worms that lay thousands of eggs daily at the venous plexus of the bladder. These eggs penetrate the bladder wall and are released into the urine to continue the life cycle [5,6]. 

Egg deposition triggers a Th2-mediated immune response characterized by eosinophilic infiltration, resulting in granulomatous inflammation, chronic fibrosis, and bladder wall calcification. Gross hematuria is the earliest and most characteristic manifestation, while dysuria, frequency, suprapubic pain, and secondary infections may also occur. In pediatric populations, the differential diagnosis of painless hematuria includes hypercalciuria, kidney stones, urinary tract infections, strenuous exercise, inherited kidney disorders such as Alport syndrome and thin basement membrane disease, glomerular diseases, and vascular abnormalities such as the Nutcracker syndrome. Chronic schistosomal infection may lead to bladder fibrosis, hydronephrosis, or squamous cell carcinoma [7]. 

The gold standard for diagnosing urinary schistosomiasis is the detection of *Schistosoma haematobium* eggs in urine sediment. PCR assays and serology offer high sensitivity and specificity, particularly in patients from non-endemic regions, although accessibility may be limited. Antigen-based tests, such as the circulating cathodic antigen and circulating anodic antigen assays, are still considered investigational. Imaging provides valuable supportive information: ultrasound may reveal bladder wall thickening or upper tract involvement, while CT urography is more sensitive for detecting calcifications or ureteric strictures [8]. Cystoscopic identification of sandy patches is highly suggestive of chronic schistosomiasis. Histopathology confirms the diagnosis by demonstrating schistosomal ova and the associated granulomatous reaction [9]. 

Reports of urogenital schistosomiasis in the UAE are extremely rare. One case was identified from an online clinical report available on a hospital website, published in May 2025, involving a 41-year-old male patient initially diagnosed with a ureteric calculus. Similar pediatric cases have been described in non-endemic European countries, often diagnosed months after the onset of symptoms, highlighting the diagnostic challenges in low-prevalence settings [3,5]. 

Praziquantel remains the treatment of choice; it induces spastic paralysis in adult worms, enabling their clearance by host immunity [10,11]. A single oral dose of 40 mg/kg achieves cure rates exceeding 85% and significantly reduces egg excretion. Early diagnosis and treatment are crucial for the prevention of complications such as bladder contracture, ureteric obstruction, and malignant transformation. Given global travel patterns, clinicians in non-endemic areas should maintain a high degree of suspicion, particularly among migrants or travelers from endemic regions [12]. Routine travel history and early cytological investigation can facilitate timely diagnosis [13]. 

## Conclusions

Bladder schistosomiasis, although rare in non-endemic countries, remains an important differential diagnosis for pediatric hematuria. Incorporating epidemiological factors into routine urological assessment is essential for early diagnosis, timely treatment, and the prevention of chronic complications such as fibrosis or malignancy. Long-term follow-up remains essential to detect and manage any delayed complications related to chronic schistosomal inflammation, and ongoing surveillance with periodic clinical and urinary evaluations is recommended to ensure sustained recovery. 
